# Supplementary material for: A Snakemake Toolkit for the Batch Assembly, Annotation and Phylogenetic Analysis of Mitochondrial Genomes and Ribosomal Genes From Genome Skims of Museum Collections
Source: Mol Ecol Resour. 2024 Oct 28;25(1):e14036. doi: 10.1111/1755-0998.14036 (PMC11646300; doi:10.1111/1755-0998.14036)
Supplement: Supplementary file 2 — Figures S1–S5 [file MEN-25-e14036-s001.docx]

**Supplemental Information for:**

**A snakemake toolkit for the batch assembly, annotation, and phylogenetic**

**analysis of mitochondrial genomes and ribosomal genes from genome skims**

**of museum collections.**

Oliver White, Andie Hall, Ben W. Price, Suzanne T. Williams, Matt Clark

**Table of Contents:**

| **Table S 1** | Page 2 |
| --- | --- |
| **Table S 2** | Page 2 |
| **Figure S 1** | Page3 |
| **Figure S 2** | Page 4 |
| **Figure S 3** | Page 5 |
| **Figure S 4** | Page 6 |
| **Figure S 5** | Page 7 |

Table S 1 Simulated taxa and reference mitochondrial and ribosomal sequences used.

Table S 2 Summary of sample quality for extracted DNA and details of factors affecting DNA including the year of sample collection, preservative (ethanol or dry shell), if shell was cracked to allow penetration of ethanol, year DNA was extracted, DNA Integrity Number (DIN) and the amplification success for four partial gene sequences (mitochondrial genes: cox1, 16S rRNA and 12S rRNA, nuclear 28S rRNA). PCR success is summarised as GenBank number if PCR were successful and published, "SEQ" if the PCR was successful and sequenced but unpublished, or "PCR only" if a band was observed when the PCR amplicon was run on a gel but attempts to sequence the amplicon were unsuccessful. Raw and quality filtered sequencing reads generated by fastp are summarised. Mitochondrial genome and ribosomal gene assembly is summarised as assembled base pairs and mapped reads. Notes on the sample filtering implemented prior to the final phylogenetic analysis are presented. Finally, a count of mitochondrial and ribosomal genes recovered are shown.


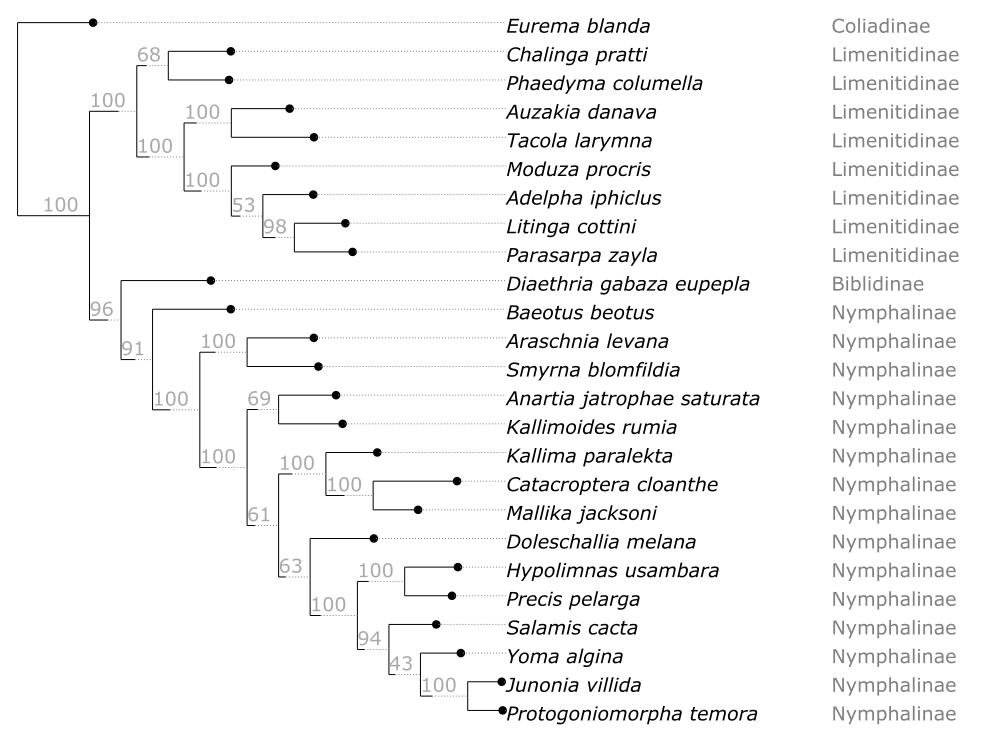


Figure S 1 Partitioned IQTREE 2 from analysis of simulated data from 25 published organelle and ribosomal genomes. The analysis included 18 genes including 13 mitochondrial protein-coding genes, two mitochondrial ribosomal genes and three nuclear ribosomal genes and visualised using ete3. The tree is rooted on the outgroup taxon *Eurema blanda* and values on branches are ultrafast bootstrap values. Tips are labelled with species and subfamily names.


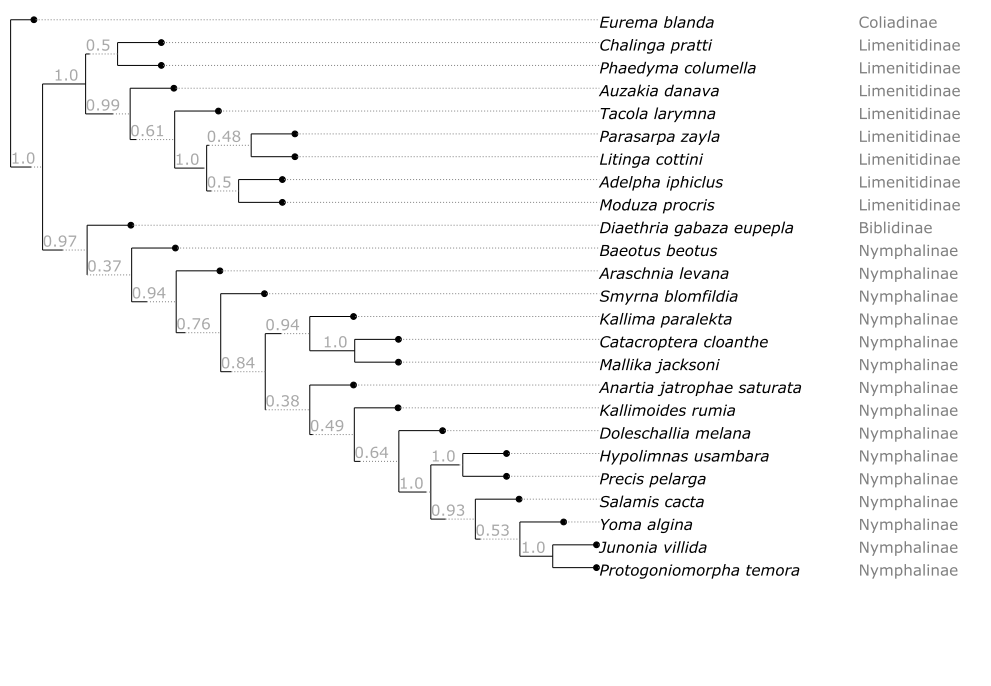


Figure S 2 Astral tree from analysis of simulated data from 25 published organelle and ribosomal genomes. The analysis included 18 genes including 13 mitochondrial protein-coding genes, two mitochondrial ribosomal genes and three nuclear ribosomal genes visualised using ete3. The tree is rooted on the outgroup taxon *Eurema blanda* and values on branches are posterior probability values. Tips are labelled with species and subfamily names.


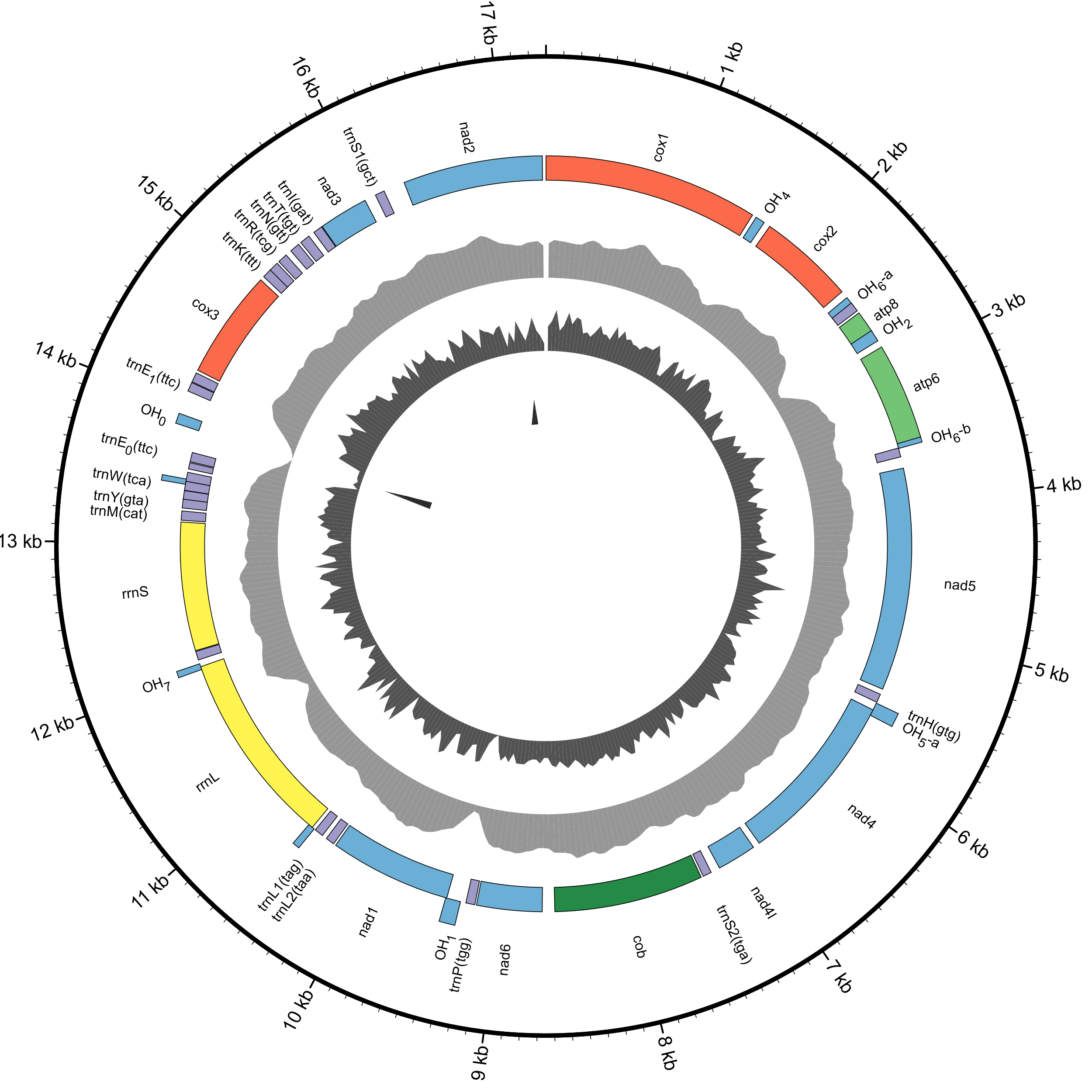


Figure S 3 Previously published assembled sequence for *Turbo cornutus* (NCBI accession NC_061024.1) with the following attributes from outside to inside: sequence position, annotation names, annotations on the + strand, annotations on the - strand, coverage (max=2779), GC content (max=0.6) and repeat content (max=1.0). This image was created using a custom organelle visualisation tool available on GitHub (https://github.com/o-william-white/circos_plot_organelle; accessed 08/2023).


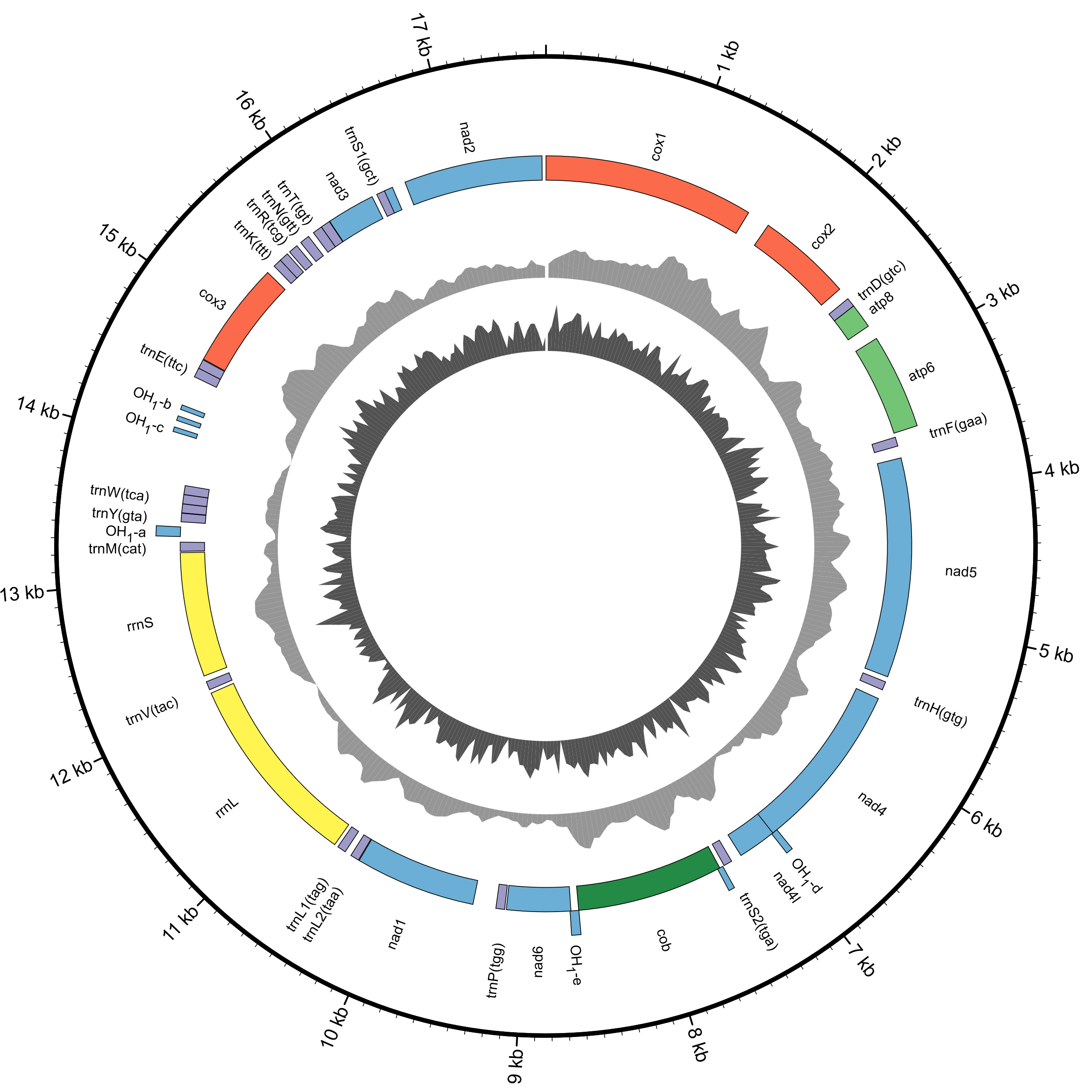


Figure S 4 Previously published assembled sequence for *Lunella* aff. *cinerea* (NCBI accession KF700096.1) with the following attributes from outside to inside: sequence position, annotation names, annotations on the + strand, annotations on the - strand, coverage (max=2779), GC content (max=0.6) and repeat content (max=1.0). This image was created using a custom organelle visualisation tool available on GitHub (https://github.com/o-william-white/circos_plot_organelle; accessed 08/2023).


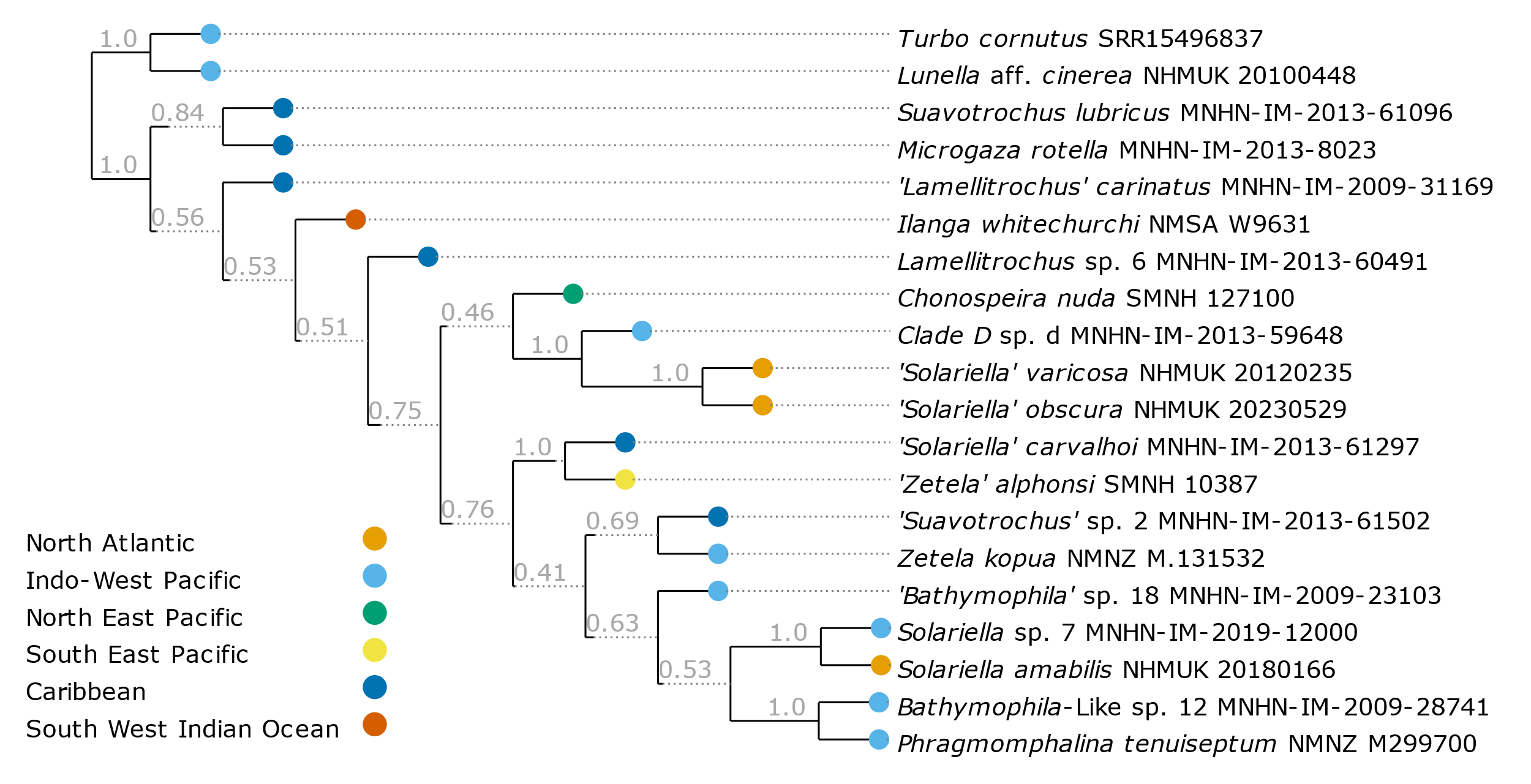


Figure S 5 Astral tree of 15 genes including 12 mitochondrial protein-coding genes, two mitochondrial ribosomal genes and one nuclear ribosomal gene (28S) and visualised using ete3. The tree is rooted on the outgroup taxa and values on branches are posterior probability values.
